# Human papillomavirus type distribution and HPV16 intratype diversity in southern Brazil in women with and without cervical lesions

**DOI:** 10.1590/0074-02760160530

**Published:** 2017-07

**Authors:** Gisele R de Oliveira, Valdimara C Vieira, Emiliana C Ávila, Fabiana Finger-Jardim, Thaís DM Caldeira, Fabiane AA Gatti, Carla V Gonçalves, Sandro G Oliveira, Vanusa P da Hora, Marcelo A Soares, Ana MB de Martinez

**Affiliations:** 1 Universidade Federal do Rio Grande, Escola de Medicina, Laboratório de Biologia Molecular, Rio Grande, RS, Brasil; 2 Instituto Nacional de Câncer, Programa de Oncovirologia, Rio de Janeiro, RJ, Brasil; 3 Universidade Federal do Rio Grande, Escola de Medicina, Centro de Obstetrícia e Ginecologia, Rio Grande, RS, Brasil; 4 Policlínica de Assistência Médica, Rio Grande, RS, Brasil

**Keywords:** HPV, lineage, HPV16, intratype variants, Brazil

## Abstract

**BACKGROUND:**

Increasing evidence suggests that human papillomavirus (HPV) intratype variants (specific lineages and sublineages) are associated with pathogenesis and progression from HPV infection to persistence and the development of cervical cancer.

**OBJECTIVES:**

This study aimed to verify the prevalence of HPV infection and distribution of HPV types and HPV16 variants in southern Brazil in women with normal cytology or intraepithelial lesions.

**METHODS:**

HPV typing was determined by L1 gene sequencing. To identify HPV16 variants, the *LCR* and *E6* regions were sequenced, and characteristic single nucleotide variants were identified.

**FINDINGS:**

A total of 445 samples were studied, with 355 from cervical scrapes and 90 from cervical biopsies. HPV was detected in 24% and 91% of these samples, respectively. The most prevalent HPV types observed were 16 (cervical, 24%; biopsies, 57%) and 58 (cervical, 12%; biopsies, 12%). Seventy-five percent of the HPV16-positive samples were classified into lineages, with 88% defined as lineage A, 10% as lineage D, and 2% as lineage B.

**MAIN CONCLUSIONS:**

This study identified a high frequency of European and North American HPV16 lineages, consistent with the genetic background of the human population in southern Brazil.

Human papillomavirus (HPV) infection is one of the main risk factors for the development of cervical cancer (zur Hausen 1976). Cervical cancer is the fourth most common type of malignancy worldwide, with approximately 90% of related deaths occurring in developing countries (Globocan 2012). According to the Brazilian National Cancer Institute (INCA 2016), this is the third most common type of cancer among women and fourth most common cause of cancer-related deaths in Brazil.

Over 200 HPV types have been identified to date, and among these, approximately 60 infect the human genital tract and can be classified according their oncogenic potential as low- or high-risk HPV types. Low-risk types are more frequently associated with genital warts and low-grade squamous intraepithelial lesions (LSIL), whereas high-risk types are more frequently associated with high-grade squamous intraepithelial lesions (HSIL) and cervical cancer (Muñoz et al. 2003, Bernard et al. 2010). HPV6 and 11 are the most prevalent low-risk types in genital warts, and HPV16 and 18 are the most frequent high-risk types found in malignant lesions and are associated with 70% of all cervical cancer cases worldwide. Nonetheless, HPV types 31, 33, 35, 45, 52, and 58 are the six most common types in the world after HPV16 and 18, and are responsible for 30% of cervical cancer cases (de Sanjosé et al. 2010).

Phylogenetic classification of HPV types is based on differences in the *L1* gene sequence, which encodes the main capsid protein, with different HPV types showing at least 10% divergence from one another (de Villiers et al. 2004). HPV types can be further classified into lineages, with genome sequences that diverge by 1-10%, or sub-lineages, with genomes that are 0.5-1.0% divergent (Burk et al. 2013).

Ho et al. (1993) classified HPV16 variants from samples from around the world using long control region (*LCR*) sequences. Four major branches, associated with geographic distribution, were identified: European-Asian (EAS) composed of European (EUR) and Asian (As) sublineages, African 1 (AFR1), African 2 (AFR2), and Asian-American (AA) lineages. A few years later, an additional lineage (North-American) was described (Yamada et al. 1997). A 2013 study by Burk and colleagues updated HPV16 lineages and sublineages using HPV16 complete genomic sequences and an alphanumeric nomenclature: lineage A for lineages previously identified as European and Asian (EAS); lineage B for African 1 (AF1), lineage C for African 2 (AF2), and lineage D for North-American and Asian-American lineages.

Interestingly, the genetic differences between HPV16 sublineages may be associated with their distinct oncogenic potentials. HPV variants may differ biologically and etiologically, leading to differences in the development and behaviour of tumours (Bernard 2002). For example, non-European variants have a general tendency to cause persistent infections and are associated with cervical lesions (Villa et al. 2000). In addition, infections with European variants that carry the T350G polymorphism, which leads to a valine replacing a leucine in the E6 protein, was suggested as an additional risk factor for persistent infection and cervical lesions (Zhang 2015). However, few reports on the distribution of HPV16 intratype variants circulating in Brazil, and especially in southern Brazil, exist. The aim of this study was to analyse the prevalence of HPV infection and the distribution of HPV types and HPV16 genetic variants in HIV-negative and HIV-positive women with or without cervical intraepithelial lesions and carcinoma and who attended centres and hospitals in southern Brazil.

## MATERIALS AND METHODS

*Study population* - A descriptive cross-sectional study was carried out in women attending the Gynecological Service of the University Hospital of Federal University of Rio Grande (HU-FURG), a regional reference centre for the treatment of HIV patients and where the Papanicolaou test was performed. Cervical scrapes were collected using a cytobrush (Vagispec^®^). Women diagnosed with HSIL in the public health system of Rio Grande city also participated in this study, with cervical biopsy samples collected at the Medical Assistance Polyclinic. Biopsy samples were collected directly from lesions. Cervical scrapes and biopsy samples were collected between March 2013 and March 2016.

Informed consent for participation in the study was obtained from all participants in strict compliance with the ethical guidelines for studies involving human subjects in Brazil as required by Resolution #466/2012 of the National Health Council. The study was approved by the Institutional Review Boards (IRB) of Federal University of Rio Grande and Prefeitura Municipal do Rio Grande (protocols # 057/2013 and 92A/2013, respectively), Brazil.

*Molecular analysis* - After collection, cervical scrapes were stored in cryogenic tubes containing 1 mL of TE buffer (10 mM Tris-HCl pH 8.5; 1 mM EDTA). Cervical biopsies were stored in tubes containing 1 mL of RNAlater®. All samples were stored at -20ºC until DNA extraction and polymerase chain reaction (PCR) amplification of HPV genomic fragments.

Cervical scrapes were centrifuged at 14,000 rpm for 15 min, supernatants were discarded, and cell pellets were used for DNA extraction. Extractions were performed using the Purelink Genomic DNA Kit (Life Technologies, Carlsbad, CA) according to manufacturer’s protocol.

For detection of HPV DNA, nested PCR was performed. The first-round PCR was performed with the external primers MY09/MY11, described by Manos et al. (1989), which amplify a 450-bp fragment of the viral *L1* region. The second round was performed with the internal primers GP5/6 (Van den Brule et al. 1990), which amplify a 150-bp internal fragment of *L1* (Table I). The reaction mixtures contained 5 µL of DNA or 3 µL of first-round product, respectively, 1× PCR buffer, 2 mM MgCl_2_, 0.5 mM dNTPs, 1 U of recombinant Taq DNA polymerase (Life Technologies, São Paulo, Brazil), 0.5 mM primers MY09/MY11 or GP5/6, and water to a final volume of 50 µL. The amplified PCR products were subjected to agarose gel electrophoresis, visualised under an ultraviolet light transilluminator, and images were captured. A positive control from a sample confirmed to be HPV-positive by sequencing, a negative control (negative by molecular and cytological assays), and a blank reaction (with no DNA) were used.

**TABLE I:** Primers and cycling conditions used for polymerase chain reaction (PCR) amplification of the *L1* region to detect human papillomavirus and the *E6* and *LCR* regions of HPV16

Primer	Sequence	Size (bp)	Cycling conditions
MY09/MY11	5’CGTCCMAARGGAWACTGATC3’ 5’GCMCAGGGWCATAAYAATGG3’	450	35 cycles of denaturation at 94ºC/40 s, annealing at 55ºC/30 s, and extension at 72ºC/1 min, and a final step at 72ºC/10 min.
GP5/6	5’TTTGTTACTCTGGTAGATAC3’ 5’GAAAAATAAACTGTAAATCA3’	150	40 cycles, with denaturation at 95ºC/40 s, annealing at 40ºC/40 s, and extension at 72ºC/40 s.
HPV16 E6 F HPV16 E6R	5’ CGAAACCGGTTAGTATAA 5’ GTATCTCCATGCATGATT	524	35 cycles with denaturation at 94ºC/30 s, annealing at 49ºC/30 s, and extension at 72ºC/45 s. Final extension at 72ºC/5 min.
HPV16 LCR *out* F HPV16 LCR *out* R	5’TGCTTGCCAACCATTCCATTGTTTTTT 5’TTGCTTGCAGTACACACATTCTAATATT	531	35 cycles with denaturation at 56ºC/30 s, annealing at 49ºC/30 s, and extension at 72ºC/40 s. Final extension at 72ºC/10 min.
HPV16 LCR *in* F HPV16 LCR *in* R	5’GTACATTGTGTCATATAAAATAAATCA 5’TGTGGGTCCTGAAACATTGCAGTTCTCTTTT	382	30 cycles with denaturation at 94ºC/30 s, annealing at 55ºC/30 s, and extension at 72ºC/45 s. Final extension at 72ºC/10 min.

PCR products were purified using an Illustra GFX PCR DNA and Gel Band Purification Kit (GE Healthcare Life Sciences, Piscataway, USA) and sequenced using an ABI Prism BigDye Terminator Cycle Sequencing Ready Reaction Kit (Applied Biosystems, Foster City, USA) with an automated ABI 3130XL sequencer (Thermo Scientific, Waltham, USA). The presence of multiple overlapping electropherogram peaks in the sequencing data were suggestive of multiple HPV infections and were not further typed, whereas clear sequences were assembled and typed. For alignment and editing of sequences and contig assemblies, SeqMan and EditSeq software of the DNA Star package (Laser Gene Inc., Madison, USA) and BioEdit Sequence Alignment Editor were used. The generated sequences were subjected to a search using the BLASTn algorithm (Available from: http://www.ncbi.nlm.nih.gov/blast/Blast.cgi) to determine HPV types based on the closest sequences in the public database.

After genotyping, HPV16-positive samples were used for the assessment of genetic variability. Nested PCR was conducted to amplify the *E6* and *LCR* regions of HPV16 with specific primers and cycling conditions shown in Table I. Reactions were performed in a final volume of 50 µL. PCR products were subjected to electrophoresis in agarose gels, as described above. Fragments were purified and sequenced using the same primers with the kit and platform described above. Sequences were assembled as described and were aligned to HPV16 reference sequences as described by Burk et al. (2013) using MEGA v.4.1. The reference sequences used can be identified by accession numbers K02718 (lineage A), AF536179 (lineage A), HQ644236 (lineage A), AF534061 (lineage A), AF536180 (lineage B), HQ644298 (lineage B), AF472509 (lineage C), HQ644257 (lineage D), AY686579 (lineage D), and AF402678 (lineage D).

To identify HPV16 variants, only samples yielding high quality *E6* and *LCR* sequences were used to identify specific single nucleotide polymorphisms (SNPs) as established by Cornet et al. (2012), which allows the identification of the lineages and sublineages proposed by Burk et al. (2013).

All DNA sequences generated in the present study were submitted to GenBank under accession numbers KY306724-KY306804.

## RESULTS

We analysed 445 samples, 355 from cervical scrapes and 90 from cervical biopsies. The average age of participants was 31 years old (SD, ± 12.8 years). Women who were subjected to cervical scrapes were 29.4 years of age, on average (SD ± 10.2 years), whereas those who had cervical biopsy samples collected were an average age of 37.9 years (SD ± 9.5 years). Of the women included in this study, 61.3% were self-described as white, 64.7% as living with a steady partner, 24.4% as having finished high school, and 37.9% as having an income between one and two minimum wages (one Brazilian minimum wage corresponds to US$ 250.00).

Among women who attended the Gynecological Service of HU-FURG, 23.6% (84/355) were HIV-1-positive, whereas all of the women who attended a public clinic were HIV-1-negative. Of the cervical scrape samples analysed, 24% (86/355) were positive for HPV DNA. The HPV prevalence among HIV-1-positive and negative women was 28.5% (24/84) and 23.2% (62/271), respectively.

Among the women who were positive for HPV infection and negative for HIV-1, LSIL were present in six samples (9.6%) and HSIL were found in one sample (1.6%). From the HIV-1-positive women, one sample (4.1%) showed HSIL.

All 86 smear samples found positive for HPV DNA were sequenced for HPV genotyping. In the HIV-1-negative samples with LSIL (n = 6), the genotypes detected were HPV6 (1/6), HPV31 (1/6), HPV33 (3/6), and HPV82 (1/6); in the sample with HSIL (n = 1), HPV16 was identified. From the HIV-1-positive women, HPV16 DNA was detected in the sample with HSIL. The prevalence of HPV genotypes in samples with negative cytological results (n = 78) from HIV-1-positive and -negative women is shown in Table II.

**TABLE II:** Human papillomavirus (HPV) genotyping of samples negative for HPV cytology from HIV-1-positive and -negative women

HPV Types	HIV-1-negative N = 55	HIV-1-positive N = 23	Total N = 78
HPV6	7 (13%)	2 (9%)	9 (14%)
HPV16	12 (21%)	7 (31%)	19 (24%)
HPV18	3 (5%)	-	3 (4%)
HPV31	2 (4%)	2 (9%)	4 (5%)
HPV33	3 (5%)	2 (9%)	5 (7%)
HPV35	1 (2%)	-	1 (1%)
HPV44	2 (4%)	1 (4%)	3 (4%)
HPV45	7 (13%)	-	7 (9%)
HPV53	2 (4%)	-	2 (2%)
HPV58	8 (14%)	3 (14%)	11 (14%)
HPV61	1 (2%)	1 (4%)	2 (2%)
HPV67	1 (2%)	1 (4%)	2 (2%)
HPV68	-	1 (4%)	1 (1%)
HPV70	1 (2%)	1 (4%)	2 (2%)
HPV82	4 (7%)	1 (4%)	5 (7%)
HPV83	-	1 (4%)	1 (1%)
HPV85	1 (2%)	-	1 (1%)

Of the 90 cervical biopsy samples analysed histopathologically, 33% (30/90) had HSIL, 33% (30/90) had LSIL, and 33% (30/90) were negative for lesions. HPV DNA was detected in 91% (82/90) of the biopsies, with all samples showing LSIL and HSIL determined HPV-positive; among women negative for histopathological lesions, the HPV prevalence was 73% (22/30), with 96% of these women infected with high-risk oncogenic HPV types.

Seventy-seven (94%) of the 82 HPV-positive biopsy samples were genotyped. In the majority of these samples (72/77), only one genotype was found; however, in five samples, multiple HPV infections were indicated by overlapping electropherogram peaks observed in the DNA sequence data. As shown in Table III, HPV16 was the most prevalent genotype detected in women both with and without squamous intraepithelial lesions (SIL) (57%); HPV58 was the second most frequent (12%).

**TABLE III:** Human papillomavirus (HPV) genotyping of biopsy samples (n = 77)

Genotype	No lesions (N = 17)	LSIL (N = 30)	HSIL (N = 30)	Total (N = 77)
HPV11	1 (6%)	-	-	1 (1%)
HPV16	10 (58%)	14 (47%)	20 (66%)	44 (57%)
HPV18	1 (6%)	6 (20%)	2 (7%)	9 (12%)
HPV31	-	2 (7%)	2 (7%)	4 (5%)
HPV33	1 (6%)	-	-	1 (1%)
HPV53	1 (6%)	1 (3%)	-	2 (3%)
HPV58	2 (12%)	4 (13%)	4 (13%)	10 (13%)
HPV70	1 (6%)	-	-	1 (1%)
Multiple infections	-	3 (10%)	2 (7%)	5 (7%)

HSIL: high-grade squamous intraepithelial lesions; LSIL: low-grade squamous intraepithelial lesions.

Of the 65 HPV16-positive samples, 49 (75%) were classified into lineages according to signatures proposed by Cornet et al. (2012). Forty-three samples (87%) were found to belong to lineage A (sublineages A1, A2 or A3). Only one (2%) analysed sequence was identified as lineage B, and lineage C was not identified in this study. Five variants (10%) were classified as lineage D (Table IV). For this analysis, we considered all SNPs in the E6 region that were proposed as criteria to classify lineages. However, in the LCR region, only two SNPs (7764C and 7786C) were used to classify HPV sequences into lineage A, and three SNPs (A7233C, A7485C, G7489A) were used to classify HPV sequences into lineage D because the amplified LCR fragments did not cover all lineage-defining SNPs in this genomic region. We failed to PCR amplify and sequence the *E6* and *LCR* regions for three and four samples, respectively.

**TABLE IV:** HPV16 lineage distribution in cervical samples according to histopathology

HPV16 lineage	Normal histology (N = 21)	LSIL (N = 12)	HSIL (N = 16)	Total (N = 49)
A	19 (90%)	10 (84%)	14 (88%)	43 (88%)
B	-	1 (8%)	-	1 (2%)
D	2 (10%)	1 (8%)	2 (12%)	5 (10%)

HSIL: high-grade squamous intraepithelial lesions; LSIL: low-grade squamous intraepithelial lesions.

Regarding demographic and clinical data for women included in the study, HPV16 lineage A was the most prevalent in women both with and without SIL. Among those infected with lineage A HPV strains, 74% of women were white and 26% were black. Among those infected with lineage D, the second most prevalent lineage in groups both with and without SIL, 60% were black and 40% were white. HPV16 lineage B was only found in one white patient with LSIL (Table IV).

Among the HPV16 lineage A-infected participants, four women carried the T350G (L83V) polymorphism in *E6*, and all lesions were classified as HSIL.

## DISCUSSION

In the present study, we determined the prevalence and genotypes of HPV, as well as HPV16 genetic variants, in HIV-negative and HIV-positive women in a major city of southern Brazil. This is the first study to describe the intratypic HPV distribution in women with and without intraepithelial lesions in this geographic area of the country.

HPV DNA was detected in 24% of the cervical smear samples analysed. A similar prevalence was found in other studies conducted in Brazil: in the north, 25.3% (Vieira et al. 2015); in the northeast, 24.5% (Fernandes et al. 2010); and in the south, 24.6% (Rosa et al. 2008). Cervical biopsies revealed the presence of HPV in all women with LSIL or HSIL. This relationship between HPV infection, cervical SIL, and cervical cancer has been well documented worldwide, since the presence of HPV is a necessary condition for the development of this neoplasm (Walboomers et al. 1999).

The biopsies analysed were from women preliminarily diagnosed with HSIL by cytological examination, and a high prevalence of high-risk HPV types was determined by PCR. However, one-third of these women were SIL-negative by histopathological analysis, showing a large difference in the specificity of these two techniques as conducted in our setting. Thus, the diagnostic triad (cytology, colposcopy, and histopathology), in association with molecular biology techniques, can be used for early identification of patients at high risk of developing cervical cancer. In fact, the American College of Obstetrics and Gynecology (ACOG) recommends the use of combined HPV tests in women over 30 years of age (ACOG 2012).

In this study, HPV16 was the most prevalent type, followed by HPV58, in women both with and without SIL. HPV16 causes approximately 70% of cervical cancers around the world and is the most prevalent type detected in samples from women with normal cytology and either low- or high-grade SIL (de Sanjosé et al. 2010, Bruni et al. 2015). The prevalence of other HPV types varies widely by geographic origin of the study population. The six HPV types most prevalent in South America are HPV16, 18, 31, 45, 33, and 58. HPV58 is the third most prevalent type in Brazil among women with either low- or high-grade SIL (ICO Information Centre on HPV and Cancer) (Li et al. 2011). In a study in Natal (northeastern Brazil), Fernandes et al. (2008) showed that HPV16 was the most prevalent genotype and that HPV58 was the second most prevalent type in women with normal cytology and LSIL; however, HPV58 showed a prevalence equal to that of HPV18 in patients with HSIL.

In addition to HPV16 and 58, types 31, 33, and 45 were also prevalent in this study in women either with or without SIL. Li et al. (2011) and de Sanjosé et al. (2010) showed through two meta-analyses that HPVs 31, 33, 45, and 58 are among the six typed most frequently reported to be associated with cervical cancer worldwide, after HPV16 and 18, but these types are not yet included in the bivalent or quadrivalent vaccines currently available in the developing world. Further studies are needed to clarify whether the eradication of oncogenic HPV16 and 18 with current vaccines can generate selective pressure, thereby increasing the prevalence of minority HPV types associated with carcinogenesis (Haug 2008). This study found a lower prevalence of HPV18 than that found in another study carried out in southern Brazil, which identified this type as the second most prevalent, followed by HPV16 (Entiauspe et al. 2014).

The association between HPV and HIV infections is well documented (Houlihan et al. 2012), with the prevalence of HPV infection higher in HIV-positive women (Corrêa et al. 2011, Menon et al. 2016). In this study, the frequency of HPV infection in HIV-positive women was also higher than that in HIV-negative women. A meta-analysis of data from various countries in North America, Africa, Asia, Europe, and South-Central America determined the prevalence and genotypes of HPV in 5,578 HIV-positive women. HPV16 was the most prevalent type in this population (Clifford at al. 2006). In Brazil, HPV16 is also the most common type detected in HIV-positive women (Corrêa et al. 2011), as we found in the current study.

In this study, the most prevalent variants of HPV16 were classified as lineage A, followed by lineages D and B. Ho et al. (1993) showed that HPV16 lineages A, B, and D are the most prevalent in populations of European, African, and Amerindian ancestry, respectively. In southern Brazil (Rio Grande do Sul), the site of this study, the population is largely of European descent, with less African and Amerindian ancestry (Manta et al. 2013). A higher prevalence of lineage A was expected and observed in our study.

In a recent meta-analysis of the worldwide distribution of HPV16 variants, lineage A was found to represent the majority of isolates and was common in all regions, except for sub-Saharan Africa and East Asia. Lineage D was also detected in all regions except for sub-Saharan Africa, whereas lineages B/C prevailed in North and Sub-Saharan Africa (Cornet et al. 2013). In Brazil, the high frequency of lineage A was also observed in women without cervical SIL in northern and southeastern Brazil (Tamegão-Lopes et al. 2014). The results by Ortiz-Ortiz et al. (2015) in southern Mexico in women with normal cytology, LSIL, HSIL, and cancer were similar to the results presented here, with an 82.1% prevalence for lineage A, 17.5% for lineage D, and 0.3% for lineage B.

HPV16 lineage D is reported to be the second most prevalent lineage after A in women with cervical SIL and invasive cancer (Vidal et al. 2016). Burk et al. (2003) reported that non-European variants were associated with a higher risk of developing cervical cancer, although much of this association was specifically related to an Asian-American variant (lineage D). In a recent study conducted by Mirabello et al. (2016), the D2/D3 sublineage was associated with increased risk of cervical intraepithelial neoplasia grade 3 and cervical cancer. In particular, D2 was associated with increased risk of glandular lesions, adenocarcinomas, and adenocarcinomas *in situ*.

The T350G variant of HPV16 lineage A was detected in the present study in women with LSIL and in women with HSIL. An earlier study suggests that this polymorphism may affect viral persistence and risk for progression to cervical lesions (Gheit et al. 2011). The E350G variant isolated in South/Central America has been associated with cervical cancer (Cornet et al. 2013), and one study reported that it has a higher capacity to transform human keratinocytes *in vitro* than the E350T variant (Sichero et al. 2012).

An important limitation of the present study was the inability to identify HPV types in co-infections. In five samples with indications of multiple infections, it was not possible to identify more than one HPV type because of the low sensitivity of the sequencing technique used (Sanger), as described by da Fonseca et al. (2016).

In conclusion, this study identified highly diverse HPV oncogenic types (HPV 16, 18, 31, 33, 45, and 82) and various HPV16 lineages in southern Brazil, which agrees with expected types based on the genetic background of this population. Studies on the prevalence of different HPV types in various regions of the world are essential to estimating the potential impact of vaccines to prevent cervical cancer currently available in the developing world and on the design of screening programs. For vaccine development, it is necessary to consider the distribution of HPV genotypes, as well as HPV intratype variants, in Brazil and worldwide, because genetic differences can determine the severity and outcome of HPV infections.
